# Mesoscale Simulation of Bacterial Chromosome and Cytoplasmic Nanoparticles in Confinement

**DOI:** 10.3390/e23050542

**Published:** 2021-04-28

**Authors:** Shi Yu, Jiaxin Wu, Xianliang Meng, Ruizhi Chu, Xiao Li, Guoguang Wu

**Affiliations:** 1 Department of Chemical Engineering, China University of Mining & Technology, Xuzhou 221116, China; 06142348@cumt.edu.cn (J.W.); meng27@cumt.edu.cn (X.M.); 4038@cumt.edu.cn (R.C.); lixiao@cumt.edu.cn (X.L.); ggwu@cumt.edu.cn (G.W.); 2 Key Laboratory of Coal-Based CO_2_ Capture and Geological Storage, Jiangsu Province (CUMT), Xuzhou 221116, China

**Keywords:** bacterial chromosome, mesoscale simulation, cytoplasmic nanoparticles, compressed cell

## Abstract

In this study we investigated, using a simple polymer model of bacterial chromosome, the subdiffusive behaviors of both cytoplasmic particles and various loci in different cell wall confinements. Non-Gaussian subdiffusion of cytoplasmic particles as well as loci were obtained in our Langevin dynamic simulations, which agrees with fluorescence microscope observations. The effects of cytoplasmic particle size, locus position, confinement geometry, and density on motions of particles and loci were examined systematically. It is demonstrated that the cytoplasmic subdiffusion can largely be attributed to the mechanical properties of bacterial chromosomes rather than the viscoelasticity of cytoplasm. Due to the randomly positioned bacterial chromosome segments, the surrounding environment for both particle and loci is heterogeneous. Therefore, the exponent characterizing the subdiffusion of cytoplasmic particle/loci as well as Laplace displacement distributions of particle/loci can be reproduced by this simple model. Nevertheless, this bacterial chromosome model cannot explain the different responses of cytoplasmic particles and loci to external compression exerted on the bacterial cell wall, which suggests that the nonequilibrium activity, e.g., metabolic reactions, play an important role in cytoplasmic subdiffusion.

## 1. Introduction

The *E. coli* bacterial chromosome, which consists of a 1.6 mm long negatively supercoiled circular DNA strand (4.6 Mbp), is subject to strong spatial restraints [1], as it needs to fit within the 2×1×1 μm ellipsoid bacterial cell wall. Since the physical organization and dynamics of bacterial chromosome play an important role in determining gene activity [2], such as DNA replication and transcription, it is of great interest to investigate how the confinement and highly compacted genome structure affect bacterial chromosomal loci dynamics. Extensive fluorescence microscope imaging results [3,4,5] reveal that chromosomal loci exhibit subdiffusion in vivo. Weber et al. [6] proposed a simple model of Rouse chain under confinement in combination with fractional Brownian motion (fBM), which suggests that the subdiffusion of chromosomal loci can be attributed to the viscoelasticity of bacterial cytoplasm.

The bacterial cytoplasm is a dense and heterogeneous medium [7], and its behavior is not fully understood. Previous particle-tracking experiments using RNA-protein particles [7] and cytoplasmic nanoparticles [8] demonstrate that cytoplasmic particles also undergo subdiffusion in bacteria and the distribution of their displacement is non-Gaussian. Moreover, Parry et al. [8] revealed that the physical properties of bacterial cytoplasm is glass-like and is fluidized by ATP-dependent cellular metabolic activities. Thus, depletion of ATP in bacterial cell has the similar effect on suppressing motions of loci [3] as well as cytoplasmic particles [8]. These experimental results imply that the subdiffusion of cytoplasmic particles and subdiffusion of chromosomal loci have the same origin, i.e., the viscoelasticity of the cytoplasm, which agrees with the predictions of free draining (Rouse) chain model developed by Weber et al. [6].

However, it has remained debatable whether the subdiffusion of loci is solely due to the viscoelastic cytoplasm, as recent simulation results [9] reveal that monomers of a simple ring-polymer decorated with side-loops can perform anomalous diffusion which agrees with experimental observations even when the cytoplasm has not been simulated explicitly in that research. More importantly, when *E. coli* bacterial cell is compressed, the motions of cytoplasmic particles have been slowed down for about one order of magnitude [10,11], while the loci subdiffusion remains almost unaffected [11]. Perturbation such as weak compression (∼5 psi) does not change the rates of cell elongation, proliferation, DNA replication, and protein synthesis significantly in *E. coli* cell [12] so that *E. coli* bacterial cells can still grow and divide under compressive force [13]. These observations suggest that the complex biological reaction networks in cell might have not been disrupted dramatically by compression. Therefore, the different responses of cytoplasmic particles and chromosomal loci to compressive force might largely be attributed to the mechanical properties of the cell, especially for short-time observations (<2 min) [11]. Hence, it is of strong interest to elucidate how the intrinsic mechanical properties of the bacterial cell, such as the viscoelasticity of cytoplasm as well as the elasticity of bacterial chromosome affect the motions of cytoplasmic particles and loci differently.

All-atom molecular dynamics (MD) simulations [14,15] allow researchers to investigate the diffusive behaviors of biological macromolecules in crowding cellular environments. Nevertheless, such all-atom MD simulations are expensive in computation. Many recent simulation studies equipped with coarse-grained model of bacterial chromosome successfully reproduce the entropic elasticity and nucleoid expansion dynamics [16,17], entropy-driven collapse/spatial organization of bacterial chromosomes [18,19,20,21,22], and macromolecular crowding effects on chromosome-arm organization in *E. coli* [23], etc. In addition, the anomalous diffusion and spatial organization of human chromosomes can be predicted by a simple polymer model incorporating a quasi-equilibrium energy landscape of human chromosomes [24]. While extensive research has been carried out to explore the slowing down of cytoplasmic diffusion resulting from the hydrodynamics and crowding effects [14,25,26], there are fewer studies on how the bacterial chromosome affects the cytoplasmic diffusion. Therefore, in this manuscript, a simple coarse-grained polymer model of bacterial chromosome (“feather-boa model”) [27,28] was adopted to simulate the motions of various loci as well as cytoplasmic particles in confinement.

As we seek to investigate to what extent the subdiffusion of loci and cytoplasmic particles can be attributed to the bacterial chromosome structure and elasticity, this manuscript focuses on the interactions between chromosome and cytoplasmic particle without simulating the components of cytoplasm explicitly. The geometry of the confinement was varied in this work to explore how the compression alters the diffusive behaviors of cytoplasmic particles. Okumus et al. [10] suggested that the external compressive force applied to the *E. coli* cells might increase the density and viscosity of the cytoplasm, for the water molecules might be expelled from the bacterial cell under compression. To examine how this volume change affects the dynamics of chromosome and particle, the monomer packing fraction (η) was also varied in our simulations. A bunch of recent research [29,30,31,32] proposed different analytical models (e.g., diffusing diffusivity) of anomalous diffusion in a heterogeneous environment, such as in bacterial cytoplasm. Those analytical models are successful in reproducing the non-Gaussianity and step distributions of cytoplasmic diffusion. Since the highly dynamic bacterial chromosome structure can also contribute to the heterogeneity of the environment in cell, hopefully, this work might shed some light on the further development of analytical models which can accurately capture the mechanical properties of bacterial cell.

## 2. Simulation Models and Methods

In this manuscript, the *E. coli* bacterial chromosome was modeled by a linear backbone chain of 200 beads which were connected by harmonic springs. Moreover, 200 side loops consisting of 40-beads were attached at every bead of the main chain, as described by D. Chaudhuri and B. M. Mulder [27,28]. The cytoplasmic nanoparticle was modeled by a single spherical bead. The bond stretching energy between consecutive beads for both backbone chain as well as 40-beads side loop was modeled by harmonic potential, as shown in Equation (1).

(1) $$U_{bond}=\frac{1}{2}k_{bond}{\left(r-\sigma \right)}^{2}$$

where $k_{bond}=100\epsilon$ is the bond energy coefficient, *r* is the distance between consecutive beads, and σ is the equilibrium bond length as well as our unit of length.

Nonbonded beads repel each other via the Weeks-Chandler-Andersen (WCA) potential [27,28], as shown in equation below:

(2) $$U_{WCA}\left(r_{ij}\right)=\left\{\begin{array}{c} 4\epsilon \left[{\left(\frac{b}{r_{ij}}\right)}^{12}-{\left(\frac{b}{r_{ij}}\right)}^{6}+\frac{1}{4}\right],\;r_{ij}\leq 2^{1/6}b\\ 0,\;\;\;\;\;\;r_{ij}>2^{1/6}b \end{array}\right.$$

where ϵ is the unit of energy, $r_{ij}$ is the distance between the $i^{th}$ bead and the $j^{th}$ bead. We choose b=σ for polymer bead pair, while we choose $b=\frac{1}{2}\sigma +r_{p}$ for polymer bead-cytoplasmic nanoparticle pair, as $r_{p}$ is the radius of cytoplasmic particle.

The interaction of all beads with the confining walls was modeled by lj93 potential, as shown in Equation (3).

(3) $$U_{wall}\left(r_{i}\right)=\left\{\begin{array}{c} \epsilon \left[\frac{2}{15}{\left(\frac{b}{r_{i}}\right)}^{9}-{\left(\frac{b}{r_{i}}\right)}^{3}\right],\;r_{i}\leq 2^{1/6}b\\ 0,\;\;\;\;\;\;r_{i}>2^{1/6}b \end{array}\right.$$

Note that b=σ for all polymer beads, irrespective of whether they belong to the backbone chain or side loops, and $b=\frac{1}{2}\sigma +r_{p}$ for cytoplasmic particle. To investigate the dynamics of both loci as well as cytoplasmic particle in bacterial cell model, Langevin dynamics method was employed to simulate this polymer model, as system temperature was fixed at $k_{B}T=1$. All the simulations in this work were implemented by the LAMMPS package [33] with time step dt=0.01 [28]. For all our Langevin dynamics simulations, the system was first equilibrated by performing a $1\times 10^{7}$ time steps long simulation. Then, another $1\times 10^{8}$ time steps simulation was performed to track the motions of cytoplasmic particle as well as loci confined by cell wall.

**Figure 1:**
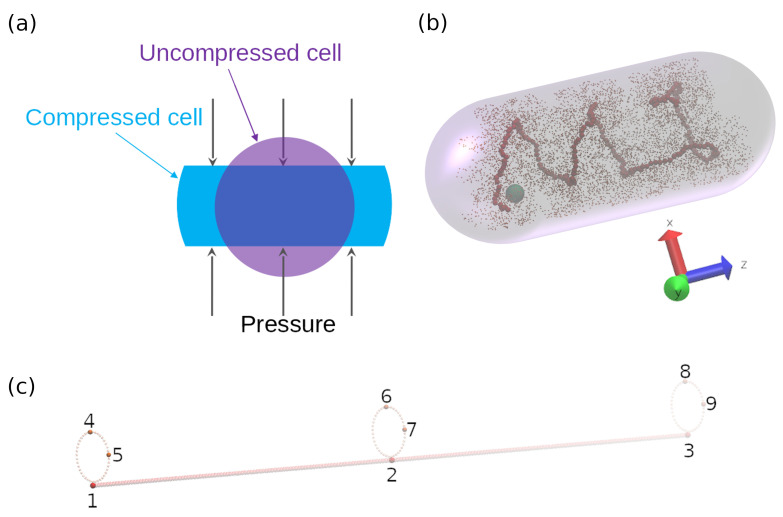
(**a**) Depiction of the cross sectional area of our model of both uncompressed and compressed bacterial cell membrane. (**b**) Snapshot of bacterial chromosome and cytoplasmic particle motion under confinement. The backbone chain, side loops, and cytoplasmic particle are represented by red beads, orange beads, and green bead, respectively. (**c**) Motions the 9 labeled beads were tracked to investigate the loci positions effects on diffusion. Note that only 3 side loops out of 200 side loops are shown.

To elucidate how the confinement geometry affects the motions of loci and cytoplasmic particle, the uncompressed bacterial cell membrane was modeled by a cylinder, while the compressed cell membrane was simulated by the intersection of a cylinder and two planes, as demonstrated in Figure 1a. Since the *E. coli* bacterial cell ends are not flat planes, a cylinder with two hemispheres of the same diameter which are attached to cylinder ends was also used as confinement for our Langvin dynamics simulations to model the ellipsoid bacterial cell, as demonstrated in Figure 1b.

The volume of the uncompressed bacterial cell model (cylinder) was varied to account for density change effects, which might contribute to the slowing-down of cytoplasmic particles motions under compression [10].

## 3. Results and Discussion

### 3.1. Subdiffusion of Cytoplasmic Particle and Chromosomal Loci in Uncompressed Cell

To compare the two-dimensional projection of 3D diffusion in our simulation results to optical microscope observations on focusing plane [11] directly, the 2D diffusion coefficient and the exponent characterizing the subdiffusion of cytoplasmic particles and loci can be fitted according to the Equation (4) as follows.

(4) $$MSD=4D_{xz}t^{\alpha }$$

where MSD is the two dimensional mean-squared displacement of cytoplasmic particle or loci within the cell, $D_{xz}$ is the 2D diffusion coefficient in *xz* plane (as shown in Figure 1b), *t* is time lag, and α is the exponent characterizing the subdiffusion. For diffusive processes, if α<1, the motion is called subdiffusive. If α>1, the diffusion is termed as superdiffusive or hyperdiffusive. For normal diffusion, α=1.

**Figure 2:**
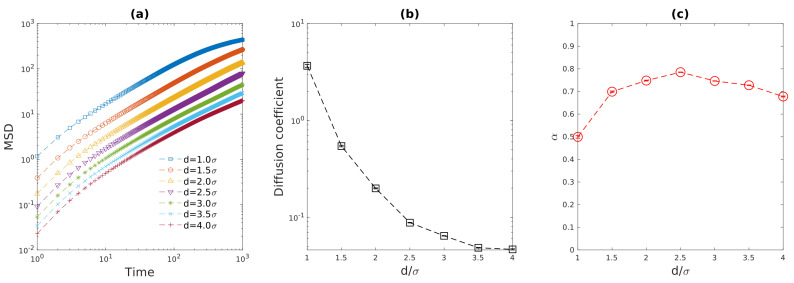
(**a**) Mean-squared displacement for cytoplasmic particles of different diameter versus time lag. (**b**) Fitted diffusion coefficients of cytoplasmic particles as functions of particle diameter. (**c**) Fitted exponent α for cytoplasmic particles as functions of particle diameter. The error bars in (**b**,**c**) represent fitted results with 95% confidence bounds.

Figure 2a shows the relationship between the 2D mean-squared displacement and time lag for cytoplasmic particles of different diameter. Exponential regression to these MSD data provides the 2D diffusion coefficients and exponent α for the particles with different sizes which are plotted against cytoplasmic particle diameter in Figure 2b,c. The narrow 95% confidence bounds for the fitted diffusion coefficient and α are represented by error bars in the figure, which indicates that the simulated MSD data agrees with Equation (4). very well. As the diameter of particle is increased from 1.0σ to 4.0σ, the diffusion coefficient of particle exhibit a ∼80-fold decrease. For the over-damped regime, the diffusion coefficient of particle in solution can be estimated by Stokes-Einstein equation $D=\frac{k_{B}T}{6\pi \mu a}$, which predicts a 4-fold decrease as a result of particle size increase. This discrepancy between our simulation results and Stokes-Einstein equation prediction can be mainly attributed to the repulsion between the bacterial chromosome and cytoplasmic particle. Since the cytoplasmic particle can collide with cell wall, it is possible that the cylindrical confinement also contributes to the slowing-down of particle motions, especially when the particle MSD approaches the square of cell wall size. As demonstrated in Figure 2c, the α for d=1.0σ particle is 0.5, which is the same as the intermediate-time scaling exponent α for a monomer on a single circular Rouse chain [6]. This result indicates that the repulsion between the so-called “feather-boa” polymer model and a spherical particle has similar effects as the correlation among circular Rouse chain monomers on particle motion.

However, for larger cytoplasmic particles (d≥1.5σ), the exponent α approximately equals to 0.7, which is similar to the experimental values (0.75±0.02) [11]. Note that for fluorescence imaging experiments, GFP-μNS nanoparticle of diameter ranging from 50 nm to 150 nm were used for particle tracking [8,11]. Thus, the diameter of such GFP-μNS particles ranges from 5% to 15% of *E. coli* cell width. As a cylinder with D=29.5σ diameter and L=50.75σ height was used to model the uncompressed bacterial cell wall in this section, the ratio between spherical particle size (d=1.5σ∼4.0σ) and cell wall width (29.5σ) ranges from 5.1% to 13.6%, which is very closed to that ratio for GFP-μNS particle in experiments. The agreement between this Langevin dynamics simulation prediction of the scaling exponent α and particle tracking results for GFP-μNS particle suggests that the repulsive force between the dynamic nucleoid structure and cytoplasmic particle allow the nucleoid to act like a heterogeneous viscoelastic “medium” or environment. Previous studies [6,7,8] suggest that the origin of cytoplasmic anomalous diffusion is the heterogeneity of cytoplasm. Since the dynamic bacterial chromosome can form “blobs”, “branch-like” structures, and cross-links in cylindrical confinement [34] or even between parallel plates [35], the bacterial chromosome also contributes to the heterogeneity of the environment that cytoplasmic particle experiences during traveling. It can be seen from Figure 2c that the simple “feather-boa” polymer model is sufficient to suppress the motion of cytoplasmic particles to subdiffusion, as the elasticity of cytoplasm is lacking in this simple model.

**Figure 3:**
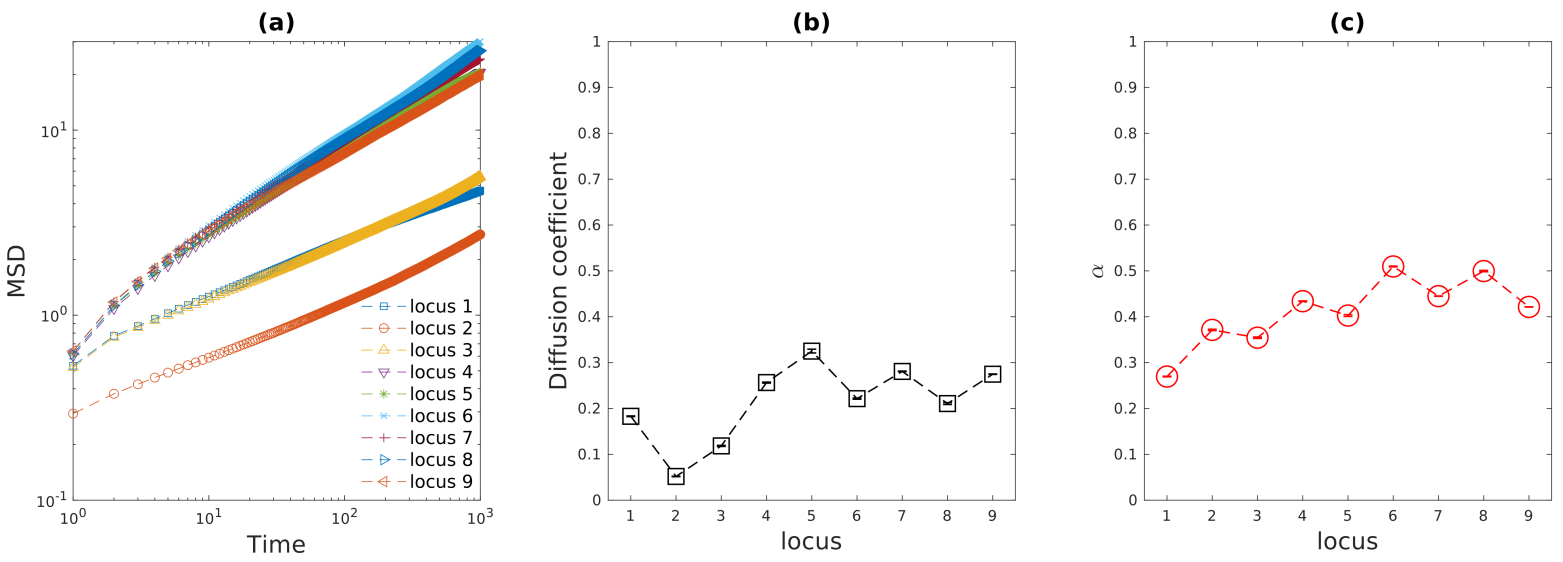
(**a**) Mean-squared displacement for loci at different positions versus time lag. (**b**) Fitted diffusion coefficients for loci as functions of locus position. (**c**) Fitted exponent α for loci as functions of locus position. The error bars in (**b**,**c**) represent fitted results with 95% confidence bounds.

The diffusive behaviors of various loci in uncompressed cell (cylinder) have also been investigated in this work. For simplicity, 9 monomers on both backbone chain and side loops were chosen to simulate the motions of chromosomal loci, as shown in Figure 1c. 2D mean-squared displacement versus time lag for those 9 chromosomal loci were plotted in Figure 3a. It can easily be seen from Figure 3a that those 9 loci can be classified into three categories. Locus 2 in the middle of the backbone chain is the slowest monomer, for the correlation between locus 2 and its neighboring monomers/side-loops is the strongest. Locus 1 and 3 at the end of backbone chain are slightly faster, since the number of adjacent side-loops are fewer and they have larger degree of freedom near the end of cell. For 4th∼9th loci, their 2D mean-squared displacements are even larger, as they are positioned on the side loops where they have large degree of freedom. The exponential regression to these 2D MSD curves allow us to obtain the 2D diffusion coefficients and scaling exponent α, which are plotted in Figure 3b,c. Again, as discussed above, the small error bars in Figure 3b,c represent the narrow 95% confidence interval, which indicates that the 2D projections of 3D MSDs of various loci agree with power law (Equation (4)) very well for the time lag up to 1000. More importantly, the fitted exponent α for loci agrees with experimental results (α≅0.4) well [4,5]. For locus 6 and 8, α≅0.5). Because these two loci are the most distant side-loop monomers away from the backbone chain, they behave more like a monomer on a single circular Rouse chain [6].

Our simulation results for loci not only agree with previous experimental studies [4,5,11], but also agree with recent simulation results carried out by P. Swain, B. M. Mulder and D. Chaudhuri [9], the research group that proposed the “feather-boa” model [28]. There are three major differences between this manuscript and the simulation performed by Swain et al. [9]: (a) Swain et al. used a circular polymer attached by side-loops instead of a linear backbone chain; (b) both circular polymer and side-loops are connected by FENE chains instead of harmonic bond (Equation (1)) in their paper; (c) Swain et al. considered crowder in cell. For both harmonic bond (Equation (1)) and FENE chain, monomers exhibit similar diffusive behaviors, suggesting that the topology of global bacterial chromosome other than the local structure of DNA is essential for chromosomal loci subdiffusion. By comparing our simulations results with results obtained by Swain et al. [9], it seems that “bottle-brush” like polymers of different structures can result in similar scaling exponent α≃0.4. Moreover, the absence of crowder in our model has negligible effect on the scaling exponent α for both cytoplasmic particles and loci in uncompressed cell. Since bacterial chromosome can associate with many NAPs and form complex structures, it is probable that the deviation between diffusive behaviors of chromosomal loci and that of circular Rouse chain monomer is caused by the elasticity of nucleoid rather than the viscoelastic cytoplasm.

### 3.2. Cell Wall Geometry Effects: Cylinder vs. Ellipsoid

**Figure 4:**
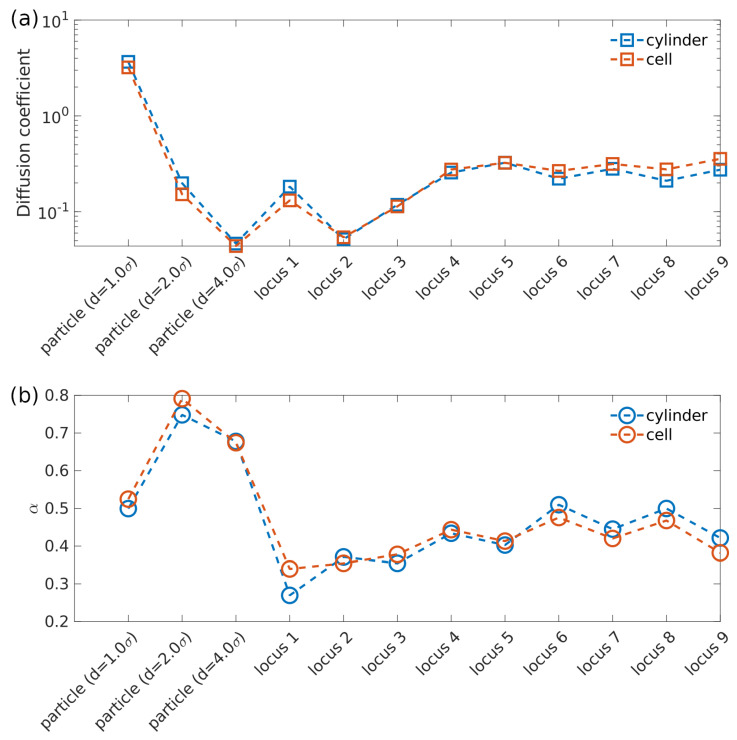
(**a**) Fitted diffusion coefficients of cytoplasmic particles with different sizes and different loci in cylinder as well as in bacterial cell shape confinement. (**b**) Fitted exponent α of cytoplasmic particles with different sizes and different loci in cylindrical confinement and in bacterial cell shape confinement.

The *E. coli* cell is an ellipsoid rather than a cylinder. It is possible that the difference between our model cell wall (cylinder) and actual *E. coli* cell wall might result in a large error of the simulated 2D projections of 3D motions of cytoplasmic particles and loci. Therefore, a cylinder attached by two hemispheres (as shown in Figure 1b) was also used as the confinement for our simulations, which is termed as cell in Figure 4 legend. The height and diameter of this cylinder is 45.83σ and 29.5σ, respectively. The diameter of the attached hemispheres is also 29.5σ. Note that the so called “cell” confinement and previous cylindrical confinement discussed in Section 3.1 have the same diameter. The monomer packing fraction η = 23.8% is the same for these two confinements.

Simulated diffusion coefficients as well as exponent α of both cytoplasmic particle and loci in confinement with different geometries are plotted in Figure 4. For both cylindrical confinement and cell-like confinement, the polymer to which closed side-loops are attached spontaneously organizes into a helicoid morphology (as demonstrated in Figure 1b), which agrees with previous research [9,27,28,36]. In other words, this confinement geometry variation has negligible effects on global structure of polymer. As can be seen in Figure 4, diffusion coefficient and exponent α only exhibit small change for both cytoplasmic particles and various loci. These observations confirm that the diffusive behaviors of particles and loci remain nearly unaffected by the change of confinement geometry as long as monomer package fraction remains the same. Thus, bacterial cell wall can be simulated by cylinder without sacrificing accuracy.

### 3.3. Density Effects on Subdiffusion of Cytoplasmic Particles and Loci

**Figure 5:**
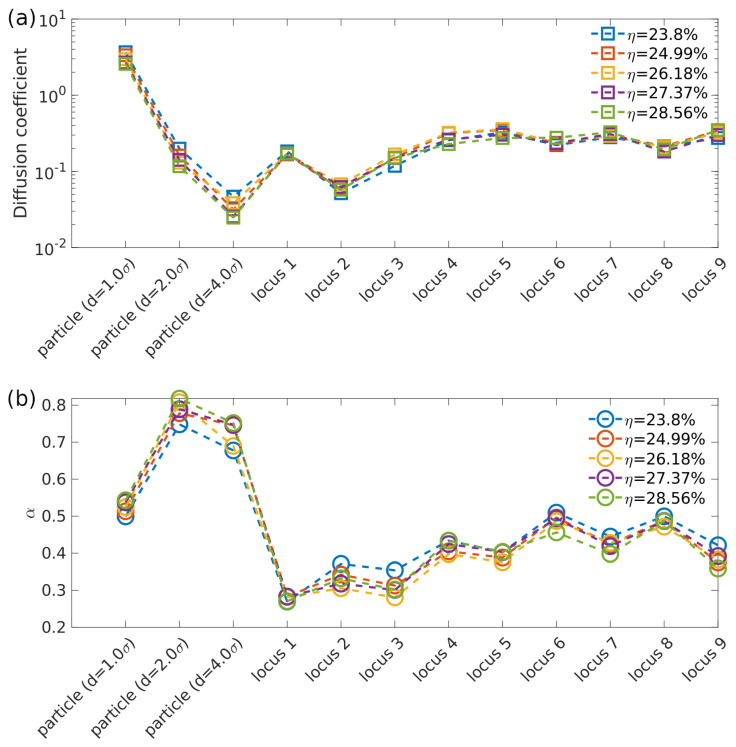
(**a**) Fitted diffusion coefficients of cytoplasmic particles of different diameter and various loci in cylindrical confinement with different sizes. (**b**) Fitted exponent α of cytoplasmic particles of different diameter and various loci in cylindrical confinement with different sizes. The η in (**a**,**b**) is monomer packing fraction.

As suggested by Okumus et al. [10], water molecules might be expelled from *E. coli* cell when compressive force is applied on the cell membrane. The bacterial cytoplasm might become denser and more viscous, as water molecules escape from bacterial cell. It is argued that this increase of cytoplasm viscosity is the origin of a significant slowing-down of cytoplasmic proteins motion which were observed by FRAP (fluorescence recovery after photobleaching) measurements [10]. To examine the effects of density change on cytoplasmic diffusion and loci motion, the diameter of cylindrical confinement was varied from 29.5σ to 26.93σ, resulting in a 20% increase of monomer package fraction η. The simulated diffusivities and α for cytoplasmic particles and loci with different monomer package fraction σ are plotted in Figure 5.

As shown in Figure 5 above, the effects of density change on subdiffusion of cytoplasmic particles with various sizes and loci are limited. As η increases from 23.8% to 28.56%, the diffusion coefficient of cytoplasmic particle decreases by a negligible fraction while the scaling exponent α increases slightly. On the contrary, the scaling exponent α for loci decreases as η increases, and becomes closer to experimental results [4,11].

Previous research [11] revealed that compressed *E. coli* cell could restore to its original shape after the compression was released. Since 20% volume reduction can cause about 8.7% reduction in cell width, i.e., about 1 pixel change of *E. coli* width under microscope [11] which has not been observed, it is unnecessary to increase monomer package fraction η any further to evaluate the density variation effects on cytoplasmic diffusion. Based on our simulation results in Figure 5, the increase of bacterial chromosome segments density, i.e., monomers package fraction η, has negligible effects on cytoplasmic particle and loci motions. While the increase of concentrations of cytoplasmic macromolecules, such as non-binding proteins, might increase viscosity of cytoplasm dramatically, it would slow-down the subdiffusion of cytoplasmic particle and loci in the same manner which is contradicted to experimental observations [11]. Therefore, cytoplasm density increase under compression is not sufficient to explain the subdiffusive behaviors of cytoplasmic nanoparticles and different loci in compressed *E. coli* cell.

### 3.4. Subdiffusion of Cytoplasmic Particles and Loci in Compressed Cell

*E. coli* bacterial cell can be compressed by PDMS membrane [10,11], and cell area on focusing plane can increase by 72% under 20 psi compression. It is extremely difficult to resolve the accurate shape of *E. coli* cell in such condition, especially considering the fact that the *E. coli* cell wall can be stiffened by stress and its elastic modulus is higher than that of PDMS membrane [37,38]. As discussed above, the simulated cytoplasmic subdiffusive behaviors remained almost the same as the cylindrical confinement was changed to “cell-like” confinement. So, for simplicity, the intersection between a cylinder and two parallel planes was used to model the compressed cell wall (as demonstrated in Figure 1a). The height of the cylinder *L*, i.e., the length of cell wall confinement, was fixed to 50.75σ, the same as in Section 3.1. The distance between the two parallel planes *H* was used to quantify the height of compressed cell, while the diameter of cylinder was the cell width. Compressed cells with three different heights 24.269σ, 19.037σ, 13.086σ were simulated, as their widths were 31.626σ, 37.5798σ, 50.15σ, respectively. Those heights and widths were chosen so as to ensure that the monomer package fraction η was fixed to 23.8%.

**Figure 6:**
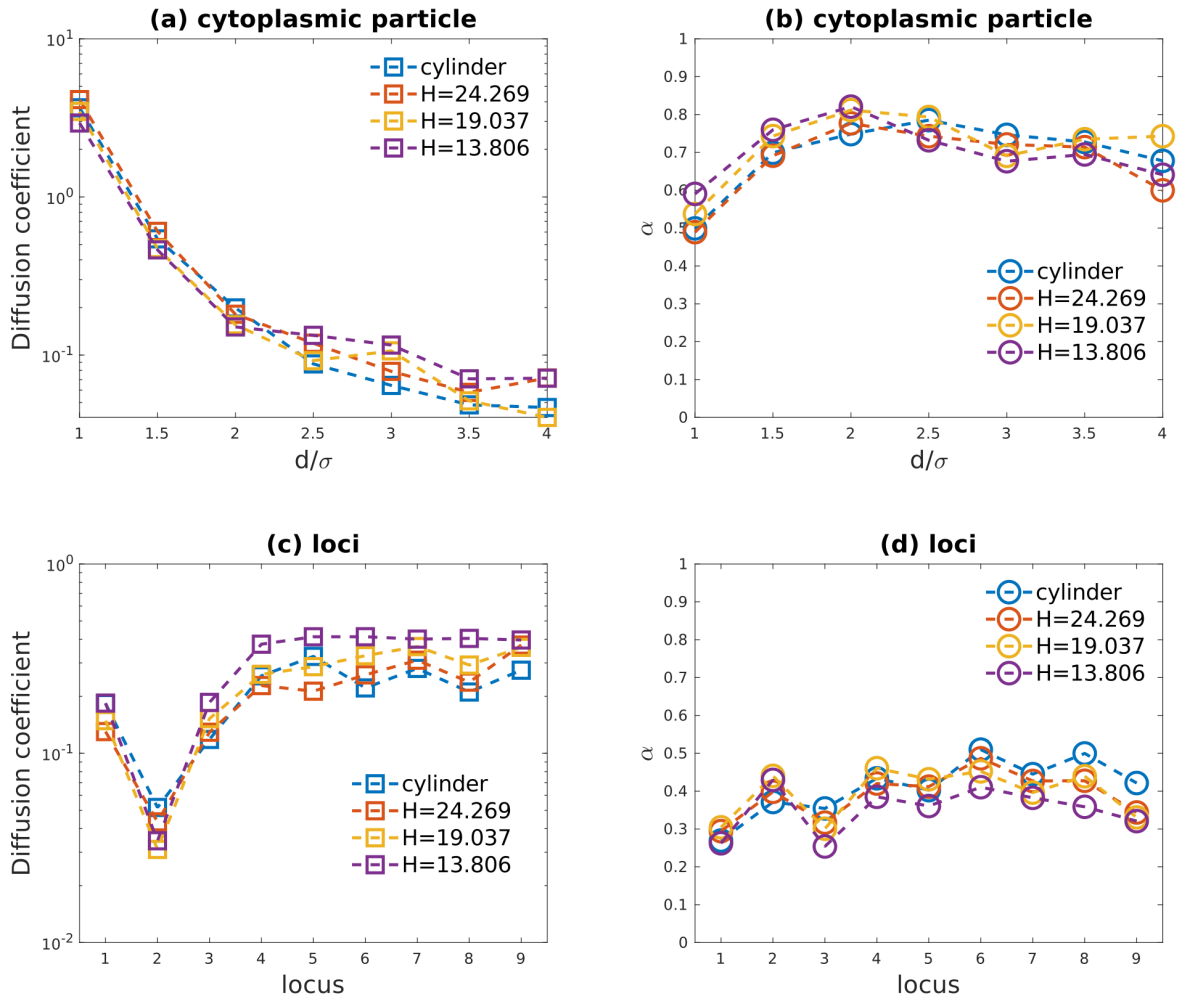
(**a**) Fitted diffusion coefficients of cytoplasmic particles as functions of particle diameter in cylindrical confinement and in compressed cell shape confinement. (**b**) Fitted exponent α of cytoplasmic particles as functions of particle diameter in cylindrical confinement and in compressed cell shape confinement. (**c**) Fitted diffusion coefficients of loci as functions of locus position in cylindrical confinement and in compressed cell shape confinement. (**d**) Fitted exponent α of loci as functions of locus position in cylindrical confinement and in compressed cell shape confinement. *H* in (**a**–**d**) is the height of the compressed cell in *y* axis.

Simulated diffusion coefficients and exponent α of cytoplasmic particles in both compressed and uncompressed cell were plotted in Figure 6a,b. For small particles (d≤2.0σ), compression decreased their diffusion coefficient and increased their scaling exponent α. On the other hand, for larger particles (d≥2.5σ), compression increased their diffusion coefficient and decreased their α. These different responses of cytoplasmic particles with different sizes to compression might be attributed to their different traveling distances, for small particles tend to scan the global heterogeneous environment within cell while large particles are likely to diffuse within a small region of cell. Nevertheless, in this work, the significant slowing-down of cytoplasmic particles in compressed cell [11] cannot be reproduced by this simple polymer model.

Simulated diffusivities and α of various loci in compressed cell as well as in uncompressed cell were plotted in Figure 6c,d. As the height of confinement *H* was decreased with increasing compressive force exerted onto bacterial cell, the diffusion coefficient of loci varied slightly, and the exponent α for loci on side-loops decreased slightly, which agrees with experimental observations [11]. With decreasing *H* and increasing cell area on focusing plane, side-loops were stretched further and collided more frequently with cell wall boundary. This might explain why scaling exponent α for loci decreased as height of confinement was reduced, especially for H=13.806σ.

While compression can alter the global structure of our model chromosome, the diffusive behaviors of monomers as well as cytoplasmic particles remained almost unaffected, according to our simulations results demonstrated in Figure 6. Because subdiffusion of particles and loci originates from heterogeneity of internal environment of bacterial cell, and only the heterogeneity from bacterial chromosome was included in this simple model, it is not surprising that there is a large discrepancy between our simulation results and experimental results [11]. In this simple model, side-loops of equal size are attached with a uniform spacing onto the backbone chain, and monomers are connected by unbreakable bonds. Therefore, the complicated bacterial chromosome reorganization as a result of compression cannot be included in this simple polymer model or in other similar models [9,36]. Importantly, since chromosomal loci and cytoplasmic particles respond to external compressive force applied onto cell wall differently, different mechanisms of cytoplasmic particles subdiffusion and loci subdiffusion seems to be necessary which cannot be reproduced by simple “bottle-brush” like polymer model.

### 3.5. Non-Gaussian Subdiffusion of Cytoplasmic Particles and Loci

**Figure 7:**
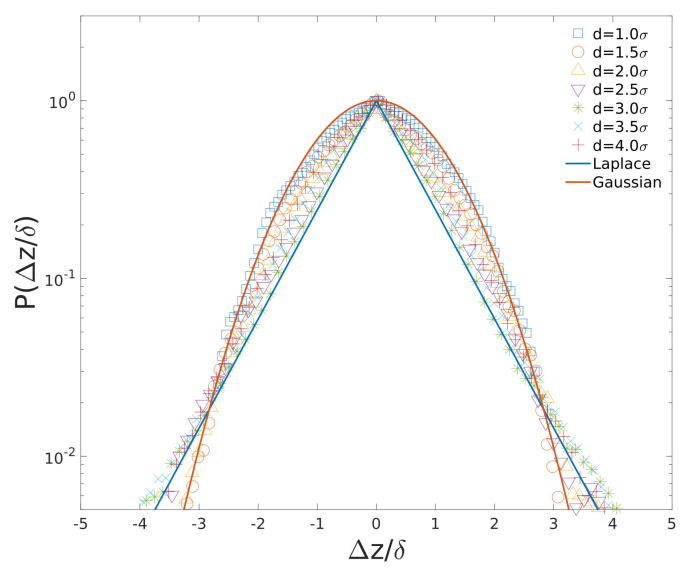
Ensemble of 1D displacement (Δz/δ) distributions of particles over time-scale ($t_{m}$ = 1000) along the long axis of cylinder. δ is the standard deviation of Δz.

In Figure 7, we plot the histograms of the 1D displacement distribution for the cytoplasmic particles with different sizes in cylindrical confinement (i.e., in uncompressed cell). The 1D displacements are defined by $\Delta z=z\left(t+t_{m}\right)-z\left(t\right)$, where z(t) is the particle position in the direction of cylinder axis (Figure 1b), $t_{m}=1000$ is the time between particle position measurements. Note that the 1D displacements of cytoplasmic particles Δz were normalized by their standard deviation δ. The probabilities for different displacements (step sizes) of different cytoplasmic particles are normalized by their maximum probabilities accordingly.

As shown in Figure 7, the Gaussian fitting curve as well as Laplace fitting curve are plotted. The probability density function of the Laplace random variable is determined by the equation below.

(5) $$P_{Laplace}\left(\Delta z;\mu ,\delta \right)=\frac{1}{\delta \sqrt{2}}exp\left(-\frac{\left|\Delta z-\mu \right|\sqrt{2}}{\delta }\right)$$

where μ and δ are mean and standard deviation, respectively. For d=1.0σ particle, the displacement distribution (blue squares) is Gaussian, since the particle diameter is the same as polymer monomer and it diffuses like a monomer on Rouse chain (α = 0.5, as shown in Figure 2c). As diameter of cytoplasmic particle increases, the 1D displacement distribution of particle changes from Gaussian to Laplacian gradually. For d=3.0σ cytoplasmic particle, the displacement distribution (green star) is nearly a perfect Laplacian distribution. For larger particles (*d* = 3.5∼4.0σ), the displacement distributions shift toward Gaussian distribution again.

Diffusing diffusivity model can be used to explain this Laplace distribution of cytoplasmic particle displacements [7,31,32], for a Laplace random variable can be represented by the product of the Gaussian random variable and the square root of the exponential random variable. Lampo et al. [7] revealed that cytoplasmic RNA-protein particle diffusivities do exhibit an exponential distribution. Therefore, a Gaussian displacement distribution of particles combined with diffusion coefficients with exponential distribution can reproduce the Laplace displacements distribution of cytoplasmic particles. For our simple “feather-boa” model, cytoplasmic particles can experience the heterogeneity of its surrounding environment as a result of randomly positioned side-loops. Hence, the diffusion coefficients of particles with intermediate sizes are diffusing, as those particles can interact with polymer segments with random configurations. For large particles (*d* = 3.5∼4.0σ), due to their slow motions, they only scan a small region of cell volume where the surrounding environment is less heterogeneous. Therefore, their displacement distributions are changing to Gaussian.

**Figure 8:**
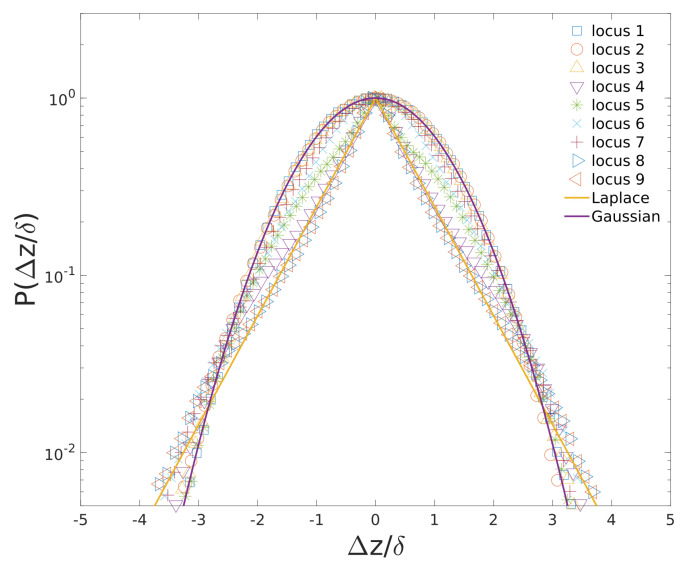
Ensemble of 1D displacement (Δz/δ) distributions of different loci over time-scale ($t_{m}$ = 1000) along the long axis of cylinder. δ is the standard deviation of Δz.

The histograms of the 1D displacement distribution for different loci in cylindrical confinement are plotted in Figure 8. As many side loops collapse around the backbone chain, the surrounding environment for backbone monomers is not as heterogeneous as that for side loops monomers (loci 4∼9). As a result, for locus on backbone chain, their displacement distributions are Gaussian. For loci 4 & 9, since these loci have large degree of freedom and are likely to experience heterogeneous environment, their displacement distributions are Laplace distribution which agrees with experimental results [11].

## 4. Conclusions

In this article, using a simple polymer model and a cylindrical confinement, the subdiffusion of both cytoplasmic particles and loci were reproduced by Langevin dynamics simulation. Previous research [3,6] suggested that such subdiffusion was due to the viscoelasticity of bacterial cytoplasm and can be captured by fractional Brownian motion (fBM) model. However, fBM model cannot explain the Laplace displacement distributions [7]. So, this manuscript provides an alternative model for cytoplasmic subdiffusion, as it not only predicts correct scaling exponent α, but also reproduces the Laplace displacement distribution of both cytoplasmic particle and loci [11]. Hence, the subdiffusion of cytoplasmic nanoparticles and loci might be a result of randomly distributed bacterial chromosome segments and the DNA elasticity.

Nevertheless, this simple polymer model failed at predicting the response of cytoplasmic particles to external compression on cell. Inasmuch as bacterial cytoplasm is a dense and spatially varied complex glass-like medium fluidized by ATP-dependent metabolic activity [8], and note that both compression and ATP depletion slow down the motion of cytoplasmic particles dramatically, it is possible that compression has disrupted the metabolic reaction network. Therefore, introducing such non-equilibrium activities (e.g., metabolic reactions) as energetic landscape rearrangement [39] into coarse-grained models similar to a “feather-boa” model might elucidate the different responses of cytoplasmic particles and loci to external compressive force.

## Data Availability

Not applicable.
